# Alternate wetting and drying irrigation maintained rice yields despite half the irrigation volume, but is currently unlikely to be adopted by smallholder lowland rice farmers in Nepal

**DOI:** 10.1002/fes3.58

**Published:** 2015-05-22

**Authors:** Katharine R. Howell, Pitambar Shrestha, Ian C. Dodd

**Affiliations:** ^1^ Lancaster Environment CentreLancaster UniversityLancasterLA1 4YQUK; ^2^ Local Initiatives for BiodiversityResearch and Development (LIBIRD)PO Box 324PokharaNepal

**Keywords:** Alternate wetting and drying, early vigor, Nepal, rice, smallholders, technology adoptiontechnology adoption

## Abstract

Alternate wetting and drying (AWD) irrigation can save water while maintaining rice yields, but in some countries its adoption by farmers remains limited. Key knowledge gaps include the effect of AWD on early vegetative vigor and its relationship with yield; the effects of AWD on yield and water use efficiency of local cultivars used by smallholder farmers; and the socio‐economic factors influencing current irrigation scheduling. To address these questions, an on‐farm field trial of dry‐season (*chaite*) rice, comparing two locally important cultivars (Hardinath‐1 and CH‐45) under AWD imposed from 1 week after transplanting to flowering and continuous flooding (CF), was carried out in Agyauli in the central Terai region of Nepal, and triangulated with social research methods exploring the rationale for current irrigation scheduling and perceptions of AWD. Although AWD plots received on average 57% less irrigation water than CF plots, yields did not significantly differ between irrigation treatments, indicating that AWD could considerably enhance crop water use efficiency in this region. In the earlier flowering, more vigorous CH‐45, there were no treatment differences in any yield component while in the later flowering Hardinath‐1, an 11% decrease in filled grain number was compensated by a 14% increase in the percentage of effective tillers per hill. Although leaf elongation rate on the main tiller did not differ between treatments, tillering and green fraction (a measure of canopy closure) were significantly higher under AWD. Surveys established that most local farmers are already using a local adaptation of AWD to modify irrigation volumes, in some cases in response to a limited and unreliable water supply. However, farmers have few direct incentives to reduce overall water use under current water governance, and formal AWD practices are therefore unlikely to be adopted despite their viability as a water‐saving irrigation technique.

## Introduction

Concerns are mounting about the production of sufficient food to feed a growing global population, due to increasingly unreliable and contested water supplies and lower overall water availability in many of the world's regions. Irrigation played a key role in the yield gains of the Green Revolution, but this was often reliant on unsustainable water withdrawals, and further yield gains on this scale therefore need to be accompanied by increases in water productivity (Rockström et al. 2007). Of particular concern is rice (*Oryza sativa* L.) production, a staple crop for over 3 billion people worldwide, which is usually either produced in water‐intensive irrigated paddy systems or is dependent on high levels of rainfall (Van Nguyen and Ferrero 2006).

To allow farmers to actively reduce their water use and associated costs, or save water for other purposes or times (Bouman et al. 2007), various water‐saving irrigation techniques have been developed. One such technology, developed in the 1990s by the International Rice Research Institute (IRRI), is alternate wetting and drying (AWD) irrigation (Price et al. 2013). As most lowland rice varieties can tolerate a 30% reduction in total irrigation volume without significantly decreasing yield (Richards and Sander 2014), this practice allows the soil to dry out partially before re‐irrigating. IRRI's “safe AWD” recommendations, intended to minimize yield reductions, are that the soil is dried until soil water depth reaches 15 cm below the surface, and that the field is re‐irrigated to a standing water depth of around 5 cm (Bouman and Lampayan 2009). Crops may be continuously flooded during and after flowering to prevent yield reductions, or AWD irrigation may be continued through this period (Bouman et al. 2007).

In field experiments and on‐farm trials, AWD significantly reduced water use whilst maintaining or even increasing yields, compared to continuous flooding (CF) (Mishra et al. 1990; Bouman and Tuong 2001; Tabbal et al. 2002; Belder et al. 2004; Mandal et al. 2009; Mishra and Salokhe 2010). The mechanisms underpinning these responses are not fully understood, but AWD has been linked to improved root growth, enabling greater access to water and nutrients at depth in the soil profile (Yang et al. 2009). Rhizosphere drying caused by AWD can also alter plant hormone signaling (Davies et al. 2011) and enhance grain filling rate, particularly in inferior spikelets (Zhang et al. 2010, 2012), thereby increasing plant water use efficiency (Tabbal et al. 2002).

However, despite these advantages, formal adoption of AWD by farmers in many rice‐producing regions has been limited (Blanke et al. 2007; Senthilkumar et al. 2009). Exploring the reasons for this is crucial to understand how AWD can be more successfully developed. A major barrier to the widespread adoption of AWD is that its effects on yield (positive or negative) vary considerably between different soils, climates, cultivars and management practices (Bouman and Tuong 2001; Price et al. 2013), as well as over time, between seasons and years (Mandal et al. 2009).

AWD may be unsuitable in sandy soils as water drains quickly and water savings are limited, whilst in heavy clays with shallow water tables AWD may be unnecessary since soil water table depth never drops below the deepest roots (Belder et al. 2004).

There may also be socio‐economic, cultural, and political reasons why farmers are reluctant to adopt new irrigation technologies (Burnham et al. 2014), especially without the scientific and visual evidence of successful local field trials (Alcon et al. 2014). Considerable research into some of the social factors determining the uptake of AWD has taken place at a regional scale and focused on economic modeling, speculative cost‐benefit analyses (Mushtaq et al. 2006; Blanke et al. 2007; Li and Li 2010; Cai et al. 2012) or analyses of the impacts of AWD adoption on farmers' income, labor costs and profitability (Moya et al. 2004).What has often been missing is a consideration of farmers' rationales for current irrigation systems, which is particularly relevant in the case of smallholders in less developed countries where factors such as land and labor may be more limiting than water or yield (Kürschner et al. 2010), farmers may be more risk averse (Koundouri et al. 2006) or water is more likely to be managed at a community level (Li and Li 2010).

Consequently, a triangulation (Nuijten 2011) of scientific and social scientific methods was identified as a suitable means to determine reasons for possibly limited adoption of AWD in Nepal. An on‐farm field trial within lowland, smallholder rice production in Agyauli Village Development Committee (VDC), Nepal, investigated local perceptions of AWD (including people's responses to the field trial) using social science research methods to determine current irrigation scheduling techniques and whether farmers need or want to change water management. Furthermore, this trial investigated the physiological effects of AWD (as opposed to CF) on early plant growth and yields of two locally grown rice cultivars, which differed dramatically in flowering date, and which were managed (with the exception of irrigation scheduling) according to local practices. In agreement with other studies, it was hypothesized that AWD would allow significant water savings without decreasing crop yields.

## Materials and Methods

**Figure 1:**
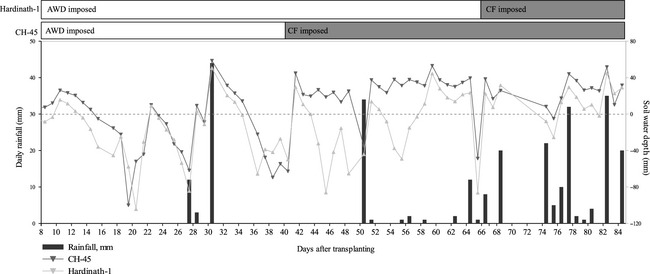
Daily rainfall in mm for Agyauli (bars) and treatment imposition of alternate wetting and drying (AWD) in two cultivars (Hardinath‐1 and CH‐45) from 8 to 84 days after transplanting. Lines show mean daily soil water depth in mm in AWD plots in the two cultivars, with dashed grey line indicating soil surface. Horizontal bars at top show periods for which AWD (white) was imposed for the two cultivars, and when they were continuously flooded (CF) during flowering (grey), in line with IRRI recommendations.

Four replications of two irrigation treatments (CF and AWD) and two locally commonly grown improved cultivars (CH‐45 and Hardinath‐1) were laid out in a randomized arrangement of 4 m x 4 m square plots, with 2 m wide irrigation channels and 1 m spacings, and earth bunds built around each plot according to local practices. The field, which was fallow over the winter, was prepared using a buffalo‐pulled plow and puddler and farmyard manure and 46 kg ha^−1^ each of diammonium phosphate and urea were applied prior to transplanting. Transplanting was carried out using local planting densities and spacings (mean number of seedlings per hill = 5, mean spacing between hills = 160 mm). The crop was top dressed with a further 60 kg ha^−1^ urea and a stemborer infestation treated with chemical insecticide at 36 days after transplanting. Heading occurred in CH‐45 and Hardinath‐1 from 32 and 51 days after transplanting, respectively, and both cultivars were harvested on Day 88. During the production cycle, mean maximum and minimum temperatures were 37°C and 26°C respectively; and mean relative humidity was 72% (meteorological data courtesy of Nepal Department of Meteorology and Hydrology). Rainfall was infrequent during the first 60 days after transplanting and more frequent thereafter as the monsoon arrived (Fig. 1).

### Irrigation scheduling

CF plots were irrigated sufficiently to maintain a mean ponded water depth of 20–30 mm, an average of twice every three days, from the local irrigation canal (*kulo)* with supplementation via buckets as necessary. AWD was imposed from 10 to 66 days after transplanting in Hardinath‐1, and from 10 to 40 days in CH‐45 (Fig. 1). The maximum water depth following rewatering of AWD plots was 78 mm above the soil surface at 30 days after transplanting, while the minimum water depth before rewatering of AWD plots was 150 mm below the soil surface at 20 days after transplanting. In line with IRRI recommendations, all plots were continuously flooded from approximately one week before, to one week after, flowering (when 50% heading was visually estimated to have been reached). IRRI recommendations for “safe AWD” (Bouman and Lampayan 2009) were adapted according to the local context. As the plow pan was approximately 15 cm below the soil surface, plots were flooded when water reached, or was just above, the plow pan. Soil water tubes (radius 52 mm) were made using cheap and readily available materials, namely plastic bottles perforated with a safety pin. Daily measurements of ponded water depth in CF plots, and soil water depth in AWD plots, were recorded to ensure adherence to these protocols. Following lodging, plots were drained, and soil moisture (but no standing water) maintained, to prevent grain germination. Irrigation volumes were calculated as a function of water depth in the inlet tube, water flow rate and duration of flooding. AWD plots received on average less than half the irrigation volume of CF plots (Table 1).

**Table 1:** Irrigation volume per unit area (m³m^−2^) applied to 20 rice plots from transplanting to harvest, under two irrigation treatments (AWD and CF), and cultivars (CH‐45 and Hardinath‐1), for five replicates for each cultivar/treatment combination. Data are means ± SE, with different letters showing significant difference to *P *<* *0.05 (ANOVA)

Treatment	Mean ± SE
AWD/CH‐45	0.86 ± 0.13 a
AWD/Hardinath‐1	0.54 ± 0.08 a
CF/CH‐45	1.6 ± 0.14 b
CF/Hardinath‐1	1.7 ± 0.33 b

### Quantifying early vigor and late lodging

Weekly manual tiller counts and daily manual measurements of the growing leaf of the main tiller of sample plants were carried out for six randomly selected border plants (to allow nondestructive access) per plot. For each leaf, its elongation rate was calculated as the difference between its lengths on subsequent days. Leaf elongation was determined to have finished when its daily elongation rate fell below 10% of its maximum, whereupon a new leaf was selected. Green fraction (GF) per plot was calculated on a weekly basis. Eight digital photographs were taken of each plot, according to the recommendations (Casadesús and Villegas 2014), and analyzed using BreedPix 2.0, which calculates the proportion of green pixels (hue 60–180°) in each image. This nondestructive technique (Lee and Lee 2013) visualized weed as well as crop growth, so fresh weed weight per plot (following manual weeding) was calculated 31 days after transplanting.

Strong winds and heavy rain associated with intense thunderstorms caused lodging in CH‐45 plots at 66, 68, and 82 days after transplanting. Following each lodging event, each plot was given a visual damage score from 0 to 5, whereby 0 represents no detectable damage, and subsequent numbers were ranked in 20% increments.

### Measuring yield components

Four days before harvest, the proportion of effective tillers was calculated from the number of tillers and panicles per sample hill. Grain size and weight were measured according to an IRRI protocol (Gomez 1972). Three random samples of four hills per plot were selected and cut three days before harvest. From each of these samples, four panicle samples were selected, and the grain from each of these stripped and stored in an envelope. The remaining grain was stripped and placed in a large envelope, and these grain samples and the straw were air‐dried for four days, after which all samples were weighed. Number of grains and number of filled grains were manually counted for each panicle sample. The remaining crop was cut at 88 days after transplanting, threshed and air dried. The grain for each plot was weighed, and moisture content estimated using a Wile 55 moisture meter, taking five moisture readings per plot. These were used to calculate adjusted grain weight at 14% moisture.

### Statistical analysis

Data were analyzed in R using quasipoisson models for time series data, and in SPSS (IBM, Portsmouth, UK) with *t*‐tests (tillering) and nonparametric tests (Kruskal–Wallis and Mann–Whitney *U*‐tests, for LER and GF, respectively) for individual days. Weed and yield data were analyzed in R with two‐way ANOVAs and general linear models, respectively.

### Social research methods

A household survey was devised and piloted with four local households. Hundred and one households were then selected at random from a list of all 321 households growing *chaite* rice in Agyauli VDC, and surveys were carried out during four weeks by the secretary of the village farmers' committee, who was identified as a suitable researcher due to his familiarity with Agyauli, fluency in Nepali and Tharu, and the respect and trust in which he is held locally (Mullings 1999; Budruk 2010). He was accompanied for the pilot surveys (by the first 2 authors of this manuscript) to provide feedback on his questioning style and ensure that he satisfied the ethical requirements of the research. The majority of respondents have limited literacy skills, so the surveys were carried out in a structured interview format. Respondents were usually interviewed at a time and location suitable to them, and were fully appraised of the purpose of the research and required to give verbal consent before the interview began.

Surveys gathered basic social information (gender, age, and farm size) followed by more specific questions about irrigation sources and management, current irrigation scheduling techniques, and irrigation‐related challenges. Respondents were also asked about their perceptions and experiences of AWD and asked to select an irrigation scheduling technique from a choice of two in a series of three choice experiments, adapting a previous model (Brouwer et al. 2009). Survey data were anonymized and translated from Nepali into English by local NGO staff (Budruk 2010). Qualitative answers were given a numerical code for analysis, and basic descriptive statistics generated using SPSS.

Follow‐up interviews were arranged to explore surprising responses about uses and perceptions of AWD. Four respondents who had said in the survey that they had used AWD were selected to give a cross‐section of ages and gender, and were interviewed when convenient to them, by fitting around their farm work. Caste and gender inequalities, although important mediators of farming practice and access to water (Panta and Resurrección 2014) were not considered here due to the focus on household‐scale experiences within a relatively homogenous ethnic group. Interviews were semistructured, with NGO staff acting as translators. Responses were recorded in writing, qualitatively analyzed and used to inform answers provided in the survey. Additionally, participant observation (Willis and Trondman 2002) of irrigation management and maintenance was carried out throughout the field trial.

## Results

### Early vigor and yield

**Figure 2:**
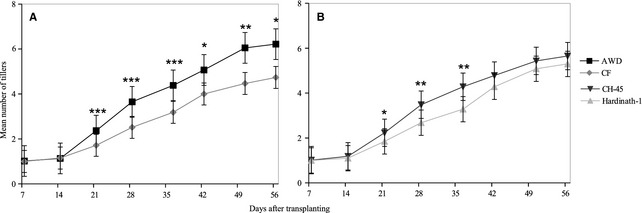
Mean tiller number, (A) Under two irrigation treatments (alternate wetting and drying, AWD and continuous flooding, CF) averaged across genotypes, and (B) In two different cultivars (CH‐45 and Hardinath‐1), averaged across treatments. Bars show ±1 standard error of 30 replicates per genotype/treatment combination. Asterisks denote statistical significance to *P *<* *0.05 (*), <0.01 (**) and 0.001 (***) (*t*‐test).

Tiller number was significantly higher under AWD than CF from 21 days after transplanting onwards (Table 2, Fig. 2A). Tiller number was significantly higher in CH‐45 from 21 to 35 days after transplanting, and remained higher thereafter.

**Table 2:** *P*‐values for the effect of irrigation and cultivar, and interaction of irrigation and cultivar (irrigation*cultivar) where including this significantly improved the fit of the statistical model, on components of early vigor and crop development. Asterisks denote statistical significance to *P *<* *0.05 (*), <0.01 (**) and 0.001 (***)

Growth characteristics	Period (days after transplanting)	*P*‐values		
		Irrigation	Cultivar	Irrigation*Cultivar
Leaf elongation rate (mm/day)	7–66	0.028*	<0.001***	Not in model
Number of tillers per plant	7–56	<0.001***	<0.001***	Not in model
Green fraction	9–53	0.020*	<0.001***	Not in model
Weed fresh weight per square meter (kg m^−2^)	31	0.002**	0.18	0.24
Lodging score	66–82	<0.001***	Not in model	Not in model

**Figure 3:**
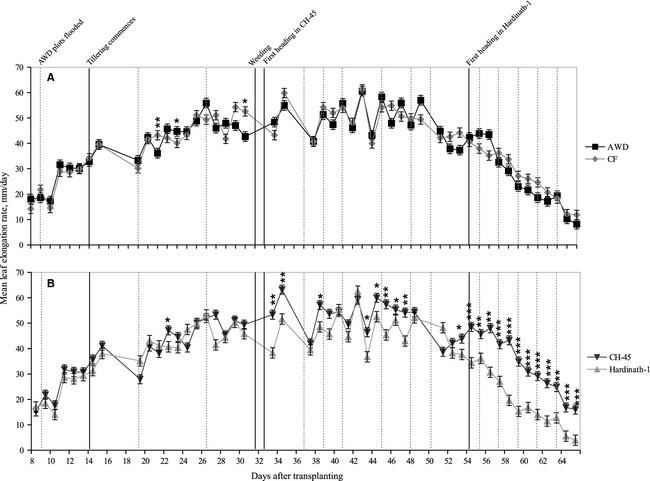
Mean leaf elongation rate, (A) Under two irrigation treatments (alternate wetting and drying, AWD and continuous flooding, CF) averaged across genotypes, and (B) In two different cultivars (CH‐45 and Hardinath‐1), averaged across treatments. Bars show ±1 standard error of 30 replicates per genotype/treatment combination. Dashed lines indicate approximate dates of flooding in AWD plots. Solid lines show key physiological and management events. Asterisks as for Figure 2 (Mann–Whitney *U*‐test).

Irrigation and cultivar significantly affected leaf elongation rate (LER) over the whole vegetative period (Table 2) and on some individual days (Fig. 3). Plants under both AWD and CF had higher mean LER at different times with no systematic pattern. CH‐45 had consistently faster rates of leaf growth than Hardinath‐1, particularly towards the end of the vegetative phase.

**Figure 4:**
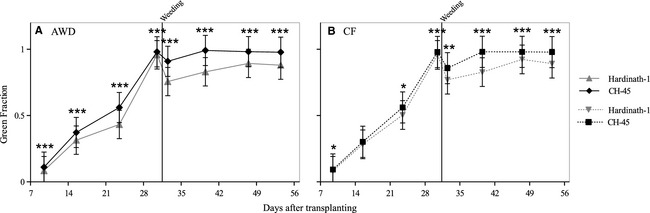
Green fraction, calculated using BreedPix analysis of eight sample digital photographs for each of 20 plots, in two different cultivars (Hardinath‐1 and CH‐45) under two irrigation regimes: (A) AWD and (B) CF. Bars show means ± 1 standard error. Asterisks as for Figure 2 (Kruskal Wallis test [AWD] and Mann–Whitney *U*‐test [CF]). Solid vertical line indicates the date of weeding.

Green fraction (GF) was significantly affected by irrigation regime, and especially cultivar (Table 2). Under AWD, CH‐45 had significantly higher GF throughout the vegetative period (Fig. 4A). Under CF, CH‐45 had significantly higher GF, but less so in the first three weeks after transplanting (Fig. 4B). Although weeding decreased GF in all treatments and cultivars, GF was less reduced by weeding in CH‐45 under both irrigation treatments. Weed weight was negatively correlated with difference in GF before and after weeding (Kendall's tau = −0.16, *P *=* *0.004). Mean GF values of nearly 1, indicating complete canopy closure, were achieved following weeding in CH‐45 under both treatments, but not in Hardinath‐1.

Weed fresh weight per plot was significantly higher, but much more variable, under CF than AWD in CH‐45, but there was no significant difference between treatments in Hardinath‐1 (Table 3). Weed fresh weight and irrigation volume were strongly positively correlated (Kendall's tau = 0.504, *P *=* *0.003). Those weeding the plots commented that weeding was slightly more difficult in AWD plots because the soil was harder. Visual lodging damage scores in CH‐45 plots were significantly higher under AWD following the second lodging event (Table 3).

**Table 3:** Weed fresh weight per square meter under two irrigation treatments (AWD and CF), and cultivars (CH‐45 and Hardinath‐1), weeded 31 days after transplanting, and visual damage scores for CH‐45 plots (Hardinath‐1 did not lodge), under the above irrigation treatments, following lodging events 66, 68 and 82 days after transplanting (dat). Data are means ± SE of five replicates, with different letters showing significant difference to *P *<* *0.05 (ANOVA)

Treatment	Weed weight (kg m^−2^)	Lodging score 66 dat	Lodging score 68 dat	Lodging score 82 dat
AWD/CH‐45	0.35 ± 0.10 a	3.6 ± 0.5 a	5.0 ± 0 b	5.0 ± 0 b
AWD/Hardinath‐1	0.45 ± 0.14 ab			
CF/CH‐45	0.69 ± 0.28 ab	2.2 ± 0.5 a	3.6 ± 0.5 a	5.0 ± 0 b
CF/Hardinath‐1	0.62 ± 0.07 b			

**Figure 5:**
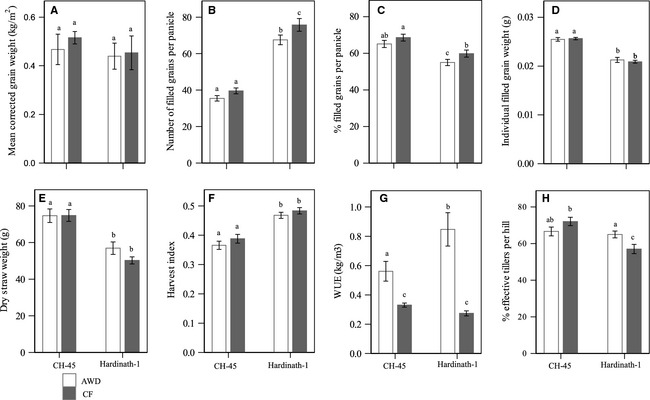
Yield components under alternate wetting and drying (AWD) and continuous flooding (CF) in two rice cultivars, CH‐45 and Hardinath‐1. Different letters show significant (*P *<* *0.05) differences between means. Bars show means ± standard error. Yield components are as follows: (A) Grain weight in kg per square metre corrected to 14% moisture (*n* = 20); (B) Number of filled grains per panicle (*n* = 240); (C) Percentage of filled grains per panicle (*n* = 240); (D) Individual filled grain weight in g, calculated using per panicle filled grain weight and number (*n* = 240); (E) Dry straw weight in g per 4‐hill sample (*n* = 60); (F) Harvest index, calculated as ratio of grain weight and total biomass weight per 4‐hill sample (*n* = 60); (G) Water use efficiency per plot, expressed in kg/m³ and calculated from corrected grain weight per plot and total volume of irrigation water applied from transplanting to harvest excluding rainfall (*n* = 20); and (H) Percentage effective tillers per hill (*n* = 120).

Grain weight per plot was not significantly different between treatments or cultivars (Table 4, Fig. 5A). The number of filled grains per panicle was significantly higher (by 89%) in Hardinath‐1, and under CF (by 12%; Fig. 5B). The percentage of filled grains per panicle was significantly higher in CH‐45 (by 18%) and again significantly higher under CF (by 6.7%; Fig. 5C). Individual grain weight was also significantly higher in CH‐45 (by 21%), but did not differ between treatments (Fig. 5D). Dry straw weight was significantly greater in CH‐45 (by 39%), although in Hardinath‐1 it was slightly higher (by 13%) under AWD than CF (Fig. 5E). Harvest index was therefore significantly higher in Hardinath‐1 (by 26%), and slightly but non‐significantly higher under CF in both cultivars (Fig. 5F). Water use efficiency (WUE), calculated as moisture‐corrected grain weight per plot divided by the total irrigation water received per plot during the whole crop cycle, was significantly higher under AWD (by 133%), particularly in Hardinath‐1 under AWD (Fig. 5G).

**Table 4:** *P*‐values for effect of irrigation, cultivar, and interaction of irrigation and cultivar (irrigation*cultivar) on yield components. Asterisks as described in Table 2

Yield components	*P*‐values		
	Irrigation	Cultivar	Irrigation*Cultivar
Corrected grain weight per square meter (kg m^−2^)	0.22	0.49	0.51
No. filled grains per panicle	0.030*	<0.001***	0.95
% filled grains per panicle	0.024*	<0.001***	0.72
Individual grain weight (g)	0.12	<0.001***	0.15
Dry straw weight per 4‐hill sample (g)	0.31	<0.001***	0.27
Harvest index per 4‐hill sample	0.21	<0.001***	0.75
% effective tillers per 4‐hill sample	0.028*	0.57	0.0056**
Water use efficiency per square meter (kg m^−3^ m^−2^)	0.0023**	0.0086**	0.0069**

There was a significantly higher percentage of effective tillers (by 2%) under AWD overall (Fig. 5H). There was a strong, significant interaction with cultivar (*P *=* *0.0056; Table 4): CH‐45 plots had a higher percentage of effective tillers than Hardinath‐1 regardless of irrigation regime. In Hardinath‐1 plots, percentage of effective tillers under AWD was significantly lower than CH‐45/CF but significantly higher than Hardinath‐1/CF.

### Social dimensions of irrigation scheduling

Survey respondents derive their irrigation either from a small shared gravity‐ fed irrigation canal, a *kulo* (55% of respondents, with *kulo*s shared between a mean number of 99 households); a shared or private shallow tubewell (36%); or a combination of *kulo* and tubewell (8%, although more farmers can access a community tubewell in times of severe water scarcity). *Kulo*s and tubewells are central to agriculture and daily life in Agyauli, as a source of irrigation, drinking and washing water and of protein such as crabs, fish and snails. Water use is charged proportionally to the area of land under irrigation, at a fixed price per season for tubewell users, and via labor or financial contributions to *kulo* maintenance for *kulo* users. Water use for both sources is regulated by community‐appointed committees.

**Figure 6:**
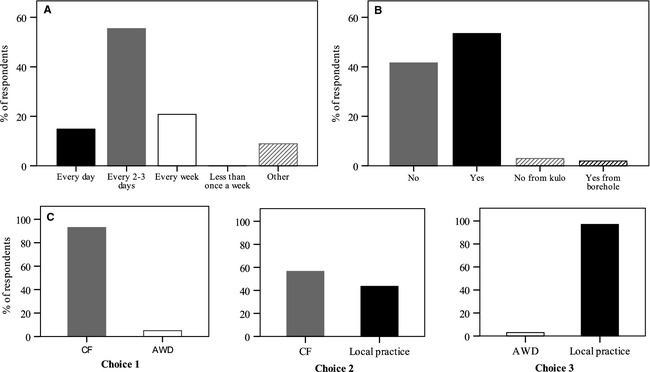
Graphs showing responses as the percentage of 101 respondents giving that response to the following questions: (A) How often do you flood the field? (B) Can you always access the irrigation water you need? (C) Choices 1, 2 and 3 were choice experiments in which respondents were asked to choose between continuous flooding (CF), alternate wetting and drying (AWD) and local practices; two respondents gave no replies, and one respondent answered “don't know” to Choice 1.

Most respondents flood their rice fields every ~2–3 days (55%). Some 15% irrigate daily, and 21% weekly (Fig. 6A), while others irrigate depending on soil type and elevation relative to water sources. Most respondents flood their field based on changes in soil water status (62%) or the availability of water (35%, particularly high amongst *kulo* users). An overwhelming 93% of respondents use the same irrigation scheduling every year. Those who do change their scheduling are influenced by interannual variability in the timings of rain and panicle development, and noted that their scheduling depends on timetabling of load‐shedding (nonavailability of electricity) and turn‐taking between farmers. Crucial times for the crop to have access to water were stated as during tillering (88% of respondents), a few days after transplanting (25%) and after weeding (14%). Respondents were aware of the need to increase the efficient use of available water by building bunds properly (74%), mending holes in them (65%) and irrigating the field carefully (12%). No respondents store water for irrigation.

More than half (56%) of respondents said they can always get access to enough irrigation water for *chaite* rice, with 5% qualifying that they can always access water from their tubewell but not the *kulo* (Fig. 6B). The weather was considered to affect water availability by 70% of respondents. This was linked to hotter and drier weather in the summer causing a decline in the water table (44%) and in natural sources of the *kulo* such as springs and rivers (49%), which in turn can affect electricity production and availability and hence the use of tubewells (3%).

When asked whether they felt their ability to irrigate *chaite* rice was restricted, the majority of respondents (63%) said that insufficient water was a problem, although 35% said it was not restricted. Other restrictions (19%) include having to wait a long time to irrigate if there is no water available during their turn, and lack of fuel or electricity for operating the tubewell, as fuel is expensive and there is extensive load‐shedding, particularly at the height of the dry season.

For respondents who cannot always access enough irrigation water, ways of preventing yield reductions comprise technical strategies, such as using an electric or diesel motor pump or earth dams in the *kulo* to ensure water reaches the field; using a tubewell to supplement their water supply (mentioned by 24% of respondents answering this question); and social strategies, such as co‐operating with and borrowing turns from other farmers (13%) and speeding up the rotation system (12%). If there is insufficient water at crucial points in crop physiological development, respondents tend to feel powerless. Some 70% said that if this occurred the crop would die or yield would be reduced. However, respondents 2 and 3, relating the experiences of a local farmer whose *chaite* rice crop is frequently destroyed by inadequate water, explained that the benefits of getting a harvest in a good year outweighs the labor and input costs of growing an ineffective crop in a water‐scarce year, particularly as straw can be used to feed livestock.

Surprisingly, given the limited formal promotion of AWD in Nepal (Uprety 2011), 91% of respondents had heard of AWD, and 90% said they had tried it on their farm. The benefits of AWD mentioned by these respondents included more effective management of pests such as stem‐borer and leaf‐roller (64%), increased tillering (27%); improved general crop health (27%); and preventing crop decay (22%). However, interview data and participant observation strongly suggested that the concept of AWD is interpreted differently by some local farmers. Interview respondents 2, 3 and 4 drain the field in response to pest infestation (stemborer and leaf‐roller), re‐irrigating the field after no more than one or two days. Respondent 1 reduces ponded water depth just before, and during, the tillering stage, which was believed to increase tillering, while increasing ponded water depth again at flowering. All four respondents said that these were well‐established practices which they had learned from older relatives.

The popularity of these long‐standing practices, and perceived need to maintain soil moisture were reflected in responses to the choice experiment questions in the survey (Fig. 6C). The overwhelming majority of respondents to these questions preferred the CF option, or flooding every 2–3 days as in local practice, over AWD with flooding every 7–10 days (95:5 and 97:3 percent respectively), whilst opinion was more divided between the former two (57:43).

## Discussion

### Early vigor is not related to final grain yield

Despite applying over 50% less water, the AWD treatment maintained a similar crop yield to CF plots (Table 4, Fig. 5A), in spite of numerous physiological changes that occurred during the growing season. Accelerated canopy closure (Fig. 4) and less frequent standing water on the soil surface under AWD (Fig. 1), coupled with partial stomatal closure during soil drying cycles (Zhang et al. 2010), likely limited soil evaporation and transpiration respectively in our experiment. Conversely, the increased GF under AWD (Fig. 4) may enhance canopy water loss early in the season, and further studies of leaf and canopy gas exchange seem necessary to reconcile these apparently conflicting findings, in explaining the lower water requirements of rice crops grown with AWD. Since there was little systematic difference in LER between irrigation treatments throughout the vegetative stage (Fig. 3) and GF following weeding (Fig. 4), AWD had a limited impact on vegetative growth except for tillering.

Contrary to expectations that soil drying (Bouman and Tuong 2001) would decrease tiller initiation and cause more frequent tiller death under AWD (Yang and Zhang 2010), tiller number was significantly higher under AWD than CF throughout development (Fig. 2A, Tables 2 and 4). Since higher tillering is often associated with higher tiller abortion later in vegetative growth (Peng et al. 1994), the higher percentage of effective tillers in Hardinath‐1 grown under AWD (Fig. 5H) is unexpected. Planting density may have a critical effect on tiller dynamics (Borzogi et al., 2011) ‘Effect of plant density on yield and yield components of rice.’ World Applied Sciences Journal. 12: 2053‐2057.) and may interact with irrigation treatment. Further work on the regulation of tiller production and survival seems necessary (Kariali and Mohapatra 2007), but the concentrations, dynamics and root to shoot ratios of different plant hormones (such as auxins, abscisic acid, and cytokinins) in response to drying and re‐ wetting cycles appear to play a role (Zhang et al. 2010; Liu et al. 2011; Dodd et al. 2015).

Although AWD delayed heading by 8 to 17 days in previous field trials (Gill et al. 2011), there were no differences in heading date between treatments, and plots under AWD had slightly, but not significantly, lower yields than CF plots. This clearly indicates the potential for AWD irrigation to be used in this and similar contexts to reduce water use without affecting yield or requiring changes in other aspects of management, but further trials are needed to account for interannual variability in effects on yield and WUE. Elsewhere in Nepal, AWD significantly decreased yields in some years (possibly due to erratic rainfall) but not others (Mandal et al. 2009). Each cultivar maintained yield under AWD in a slightly different way: with no significant variation in yield components in CH‐45 which was exposed to a shorter duration of AWD (Fig. 1), while more effective tillers in Hardinath‐1 compensated for a decrease in grain number (Fig. 5). Significantly higher numbers of tillers and effective tillers under AWD may have caused more competition between tillers and panicles for plant resources, resulting in significantly lower grain weight, number and filling (Peng et al. 1994). This contrasts with previous findings that lower tiller number under AWD was compensated for by higher grain weight and a greater proportion of grain filling per panicle (Bouman and Tuong 2001; Zhang et al. 2010).

There were also significant interactions between irrigation treatment and cultivar (Table 4). Despite much earlier heading of CH‐45 (Yoshida 1981), higher grain weight and filling occurred, perhaps related to the slower decline in LER in this cultivar (Fig. 3). GF was significantly higher in CH‐45 throughout the vegetative period under AWD, probably related to its enhanced shoot vigor as a pre‐IR8 (a widely released semidwarf) variety, which may contribute to its greater weed competitiveness. There was also an apparent trade‐off between cultivars between grain weight and filling, and number of grains. The resource trade‐off between grain weight and number in cereals is well‐established (Gambín and Borrás 2010), but a study in longer duration rice varieties found no significant relationships with grain filling rate, concluding that this is genetically determined (Venkateswarlu et al. 1981).

The relationship between early vigor and yield components is complex. There appears to have been a trade‐off between tillering, and panicle size and grain development, as found previously (Peng et al. 1994). This greater early vigor in CH‐45 significantly increased dry straw weight and significantly lowered harvest index, but had no effect on overall grain yield. Interestingly, both genotypes and irrigation treatments produced similar yields regardless of differences in early vigor.

AWD has been proposed to increase resilience to lodging, since the crop may develop deeper roots to access soil water (Yang et al. 2009). However, in heavy soils such as the clays in Agyauli, drying can lead to particle cementation (Rao and Revanasiddappa 2006) and soil compaction (Sanchez 1973). This may restrict root growth and render a crop under AWD more vulnerable to lodging, as observed here (Table 3). Whether limited root development enhances tillering but slows grain development under AWD (Figs. 2 and 5) merits further studies.

Although more weeds have been reported (but not quantified) in on‐farm trials of AWD (Kürschner et al. 2010), in this experiment the opposite occurred. This could be related to the relatively shallow ponded water depths in CF plots (20–30 mm) and to soil contraction under AWD restricting weed growth as well as crop root development. Enhanced crop tillering can decrease weed competitiveness (Zhao et al. 2006) but the choice experiment and conversations with local farmers suggest that weed competitiveness is not considered important in selecting cultivars and irrigation scheduling. Weed weight was negatively correlated with the difference in GF calculated immediately before and after weeding (Kendall's tau = −0.16, *P *=* *0.004), indicating that the accuracy of BreedPix in measuring crop development would benefit from detecting the color and texture of weeds in digital images (Gée et al. 2008; Meyer and Neto 2008).

### From promising science to a socially viable agronomic technique?

The agronomic results, when combined with the findings that many local farmers experience problems accessing sufficient water to irrigate *chaite* rice (Fig. 6B), suggest that AWD shows considerable promise. However, although respondents are familiar with the concept of AWD and may even have tried it, they overwhelmingly said that they would be unwilling to adopt AWD as their principal irrigation scheduling technique. Determining the reasons for this provides insight into the wider challenges of promoting the adoption of particular agricultural technologies, and of pursuing science and technology developments which will most benefit farmers.

AWD, as recommended by IRRI, relies on two key assumptions about the socio‐economic context in which it is to be adopted. Firstly, that adopting AWD is a straightforward and rational choice for farmers, as long as water supply is reliable and can be controlled separately for each field or farm. Secondly, that there are strong, immediate incentives for reducing on‐farm water consumption, for example if water is paid for per volume per farmer (Moya et al. 2004; Blanke et al. 2007). However, in Agyauli and many other regions in South and East Asia (Mushtaq et al. 2006; Kürschner et al. 2010), one of the main problems facing irrigated agriculture, and for the adoption of AWD, is unreliability, which could result in soil drying beyond the “safe AWD” limits at key points in crop development, or require more frequent, pre‐emptive flooding, thereby reducing possible water savings. Likewise, many farmers pay for water by land area, not by volume, and use a shared system, for example with a rotation system determined by a users' committee. Thus they have little direct incentive to save water, and limited control over timing, depending on the flexibility of the system and politics within the users' committee (Meinzen‐Dick and Zwarteveen 1998). Furthermore, in this region, water leaking from one field into another may contribute to other farmers' crops, to other water uses or the health of the whole agro‐ecosystem, so more water than the actual crop requirement may be applied (Moya et al. 2004).

Research on AWD adoption has recognized and addressed some of these problems, primarily advocating changes to water governance mechanisms like stricter water pricing, and the creation of infrastructure such as storage ponds (Mushtaq et al. 2006; Li and Li 2010). It also focuses on education, dissemination and uptake at a variety of scales (Lampayan et al. 2015). However, context is important, and recommended policy, pricing or infrastructural fixes are rarely simple in practice, since farmers are rarely able to respond only to stable legal and economic factors (Berry 1993). Although agricultural practices may not be optimized due to rapid and externally driven change, they are usually contextually embedded. This means they are adapted to and implicated in a wide range of political, social, cultural and economic practices and institutions at different scales, as well as ecological and infrastructural conditions (see, for example, Berry 1993; Lansing 2006). Consequently, apparently simple changes to agricultural practices, such as AWD, may in fact have wider impacts and necessitate wider changes (Lansing 2006; Burnham et al. 2014). In Agyauli, for example, irrigation scheduling is socially and politically mediated, for example the management of water by *kulo* committees and of electricity by central government. It is linked to cultural and social factors including familiarity with particular techniques, labor availability and ease of weeding. As in previous field trials in Nepal, the impacts of AWD on soil properties take on new significance when part of a (rice‐wheat) rotation system (Mandal et al. 2009). Further agronomic trials exploring the short‐ and long‐term effects of AWD on pest and weed populations and soil properties would help ensure a holistic view of proposed changes to agricultural practices. Some of the features of AWD, such as its use of a simple and affordable field water tube, have promoted its large‐scale uptake in other rice‐growing areas such as the Philippines, Vietnam and Bangladesh (Lampayan et al. 2015). However, Nepal is a different context, especially in terms of water governance, irrigation infrastructure and agricultural extension services, and as such will require a different approach to promoting AWD.

Agricultural anthropologists such as Richards (1989) have long called for agricultural and agronomic research to consider farming as a performance: a lived process in which change is determined by dynamic environmental, cultural and social factors as well as economic costs and benefits. Additionally, the use of and changes to agricultural practices are dynamic. Richards argues that farming happens, unlike a predesigned field trial, “in time”, with decisions and strategies made and developed in response to changing conditions, such as weather patterns and labor availability. Similarly, in Agyauli, irrigation scheduling happens *in time*. Although farmers generally use the same irrigation scheduling each year, the day‐to‐day practice of irrigation depends on various factors, such as weather, water availability, local water governance, neighbors' irrigation requirements and how level the field is. Carrying out an on‐farm irrigation field trial, using the novel method of simultaneous experiment and participant observation, provided a lens onto day‐to‐ day agricultural practice, enabling a better understanding of the potential suitability of AWD to this setting.

The contextual embeddedness of agricultural practices raises two interlinking, crucial but often neglected considerations for researchers working on new agricultural science and technologies: understanding a particular agricultural context through interdisciplinary research, and involving farmers, and their knowledge, opinions and needs, through participatory research and extension such as farmer to farmer training and farmer field schools (Fortmann 2008). There are growing concerns that the urgency and global focus of current food security narratives are marginalizing such approaches (Tomlinson 2011), despite the fact that locally specific, farmer‐driven innovation may hold many of the answers to food security challenges (MacMillan and Benton 2014). Despite the wealth of detailed social science research into agricultural systems, and resilience and change within them, these understandings are rarely effectively integrated with conventional agricultural science research, with major implications for the relevance of research findings to the realities of farming communities (Cornwall et al. 1994; Giller et al. 2011). This paper offers an example of one form of interdisciplinary research which can help to incorporate social and physiological understanding of agronomic techniques. Major progress has been made in making AWD accessible and appropriate for farmers elsewhere in Asia (Lampayan et al. 2015); and more context‐specific, interdisciplinary and participatory research could help adapt it for other rice‐ producing regions such as Nepal.

## Conclusions

AWD proved to be an extremely effective water‐saving irrigation technology in the context of *chaite* rice production in Agyauli VDC in lowland Nepal, greatly increasing the water use efficiency of locally important varieties without decreasing yields. Both irrigation treatment and cultivar affected early vigor, but had no effect on final yield. However, it is unlikely to be adopted in Agyauli and areas with similar socio‐economic conditions unless local water management, and use (or both) is altered. Future extension work on AWD needs to consider ways of supporting farmers in changing water management and governance, but also ways in which the technology and the science underpinning it could be adapted for these contexts. Increasing the involvement of farmers in AWD research, through the use of participatory and interdisciplinary methods, could improve uptake of these or similar water‐saving techniques.

## Conflict of Interest

None declared
